# Unveiling the Role of the Integrated Endoplasmic Reticulum Stress Response in *Leishmania* Infection – Future Perspectives

**DOI:** 10.3389/fimmu.2016.00283

**Published:** 2016-07-22

**Authors:** K. L. Dias-Teixeira, R. M. Pereira, J. S. Silva, N. Fasel, B. H. Aktas, U. G. Lopes

**Affiliations:** ^1^Institute of Biophysics Carlos Chagas Filho, Federal University of Rio de Janeiro, Rio de Janeiro, Brazil; ^2^Institute of Microbiology Paulo de Goes, Federal University of Rio de Janeiro, Rio de Janeiro, Brazil; ^3^Department of Biochemistry and Immunology, University of São Paulo, Ribeirão Preto, Brazil; ^4^Department of Biochemistry, Faculty of Biology and Medicine, Center for Immunity and Infection Lausanne, University of Lausanne, Lausanne, Switzerland; ^5^Laboratory of Translation, Department of Hematology, Brigham and Women’s Hospital, Harvard Medical School, Boston, MA, USA

**Keywords:** *Leishmania*, ER stress, XBP-1, IFN-1, PERK, ATF4

## Abstract

The integrated endoplasmic reticulum stress response (IERSR) is an evolutionarily conserved adaptive mechanism that ensures endoplasmic reticulum (ER) homeostasis and cellular survival in the presence of stress including nutrient deprivation, hypoxia, and imbalance of Ca^+^ homeostasis, toxins, and microbial infection. Three transmembrane proteins regulate integrated signaling pathways that comprise the IERSR, namely, IRE-1 that activates XBP-1, the pancreatic ER kinase (PERK) that phosphorylates the eukaryotic translation initiation factor 2 and transcription factor 6 (ATF6). The roles of IRE-1, PERK, and ATF4 in viral and some bacterial infections are well characterized. The role of IERSR in infections by intracellular parasites is still poorly understood, although one could anticipate that IERSR may play an important role on the host’s cell response. Recently, our group reported the important aspects of XBP-1 activation in *Leishmania amazonensis* infection. It is, however, necessary to address the relevance of the other IERSR branches, together with the possible role of IERSR in infections by other *Leishmania* species, and furthermore, to pursue the possible implications in the pathogenesis and control of parasite replication in macrophages.

## Introduction

The endoplasmic reticulum (ER) is a dynamic tubular network involved in different processes such as protein folding, lipid synthesis, and the biogenesis of autophagosomes and peroxisomes (1). When the process of protein synthesis and/or folding is disturbed, the ER induces a transcriptional program, the integrated endoplasmic reticulum stress response (IERSR), leading to the increase of ER-chaperone expression, lipid synthesis, and the induction of other sets of gene products involved in the retrograde transport and degradation of unfolded proteins (ERAD) (2). These conserved adaptive responses reduce demand on the folding capacity or ER, increase ER’s folding capacity, and clear this organelle off of unfolded proteins. However, during this process, a set of genes that also regulate the expression of cytokines and promote the resistance to oxidative stress are upregulated. The three branches that regulate the ER response are comprised by the activating transcription factor 6 (ATF6), inositol-requiring kinase 1 (IRE-1), and the protein kinase R (PKR)-like endoplasmic reticulum kinase (PERK). IRE-1 activates the X-box binding protein 1 (XBP-1), a transcriptional factor that plays a critical role in cellular homeostasis and regulates the expression of important cytokines related to the anti-viral immunity response, such as IFN1-β. PERK phosphorylates eIF2α, which reduces overall protein synthesis while upregulating the expression of activating transcription factor 4 (ATF4), which drives the expression of genes that play a critical role in restoring cellular homeostasis, resistance to oxidative stress together with genes related to the autophagic pathway and the innate immunity response. Interestingly, both XBP-1 and ATF4 can be activated by toll-like receptors (TLRs). For instance, the engagement of TLR2 and TLR4 can specifically activate XBP-1 leading to the production of pro-inflammatory cytokines that restrain bacterial burden in infected macrophages (3). ATF4 can be directly activated by the TLR4-MyD88 pathway following stimulation of human monocytes with lipopolysaccharide (LPS) (4).

Viruses can selectively induce specific branches of the IERSR. For instances, human cytomegalovirus and hepatitis C activate the IERSR response, while some viruses, such as dengue virus and hepatitis C virus induce the IERSR trough the exploitation of the ER membranes during the replication process (5). Additionally, some viruses induce the IERSR and the inhibition of the translational process due to the phosphorylation of eIF2-α, reducing the production of cytokines and interfering with the host immune response. This process is highly induced by enteroviruses (6). Some viruses adapted the IERSR pathways to favor their infection directly. The phosphorylation of eIF2-α induces the translation of a specific set of proteins including ATF4. ATF4 can, for example, enhance human immunodeficiency virus (HIV) replication through a synergistic interaction with the HIV regulatory protein Tat (7).

The role of IERSR pathways in parasite infection is poorly investigated. Recently, it was reported that *Plasmodium berghei* induces the ER stress response and XBP-1 mRNA splicing and translation of the transcriptionally active XBP-1 spliced form (XBP-1*s*) in hepatocytes. This activation was demonstrated to be crucial for parasite replication inside hepatocytes and to the progression of the infection (8). XBP-1*s* can modulate the synthesis of phospholipids, such as phosphatidylcholine (PC), in hepatocytes. PC is a major component of membranes, and it has been demonstrated that malaria parasites uptake host-derived PC and, most probably, PC is also employed for enlarging the parasitophorous vacuole membrane (9). Most recently, we showed that induction of ER stress favors *Leishmania amazonensis* infection in a TLR2-dependent manner, culminating in the formation of XBP-1*s*. XBP-1 induces IFN-β expression and modulates the oxidative response of infected macrophages, thereby promoting parasite proliferation (10).

However, it will be important to test these observations in other *Leishmania* species and to address the relevance of the PERK/ATF4 and ATF6 branches of the IERSR during *Leishmania* infection.

## The Role of XBP-1 in *Leishmania* Infection

We recently observed that *L. amazonensis* induces the activation of XBP-1 in macrophages. RAW 264.7 cells knocked down for XBP-1 exhibited reduced parasite load, likely due to impaired translocation of the IRF3 transcription factor resulting in reduced IFN-1 expression (10). We also observed that infected XBP-1 knocked down macrophages produce higher nitric oxide levels and reduced Hemeoxygenase (HO)-1 expression compared to control macrophages. However, how XBP-1 controls oxidative stress in *L. amazonensis* infection requires further investigation. One mechanism that could induce this effect is the activation or repression of the NF-κB transcription factor. *L amazonensis* activates an NF-κB p50/p50 repressor homodimer, which promotes reduction in iNOS expression and favors parasite growth (11). The production of ROS can activate the ER stress response, which can suppress NF-κB activation in the later phase of IERSR (12). The protein A20, an ubiquitin-editing NF-κB inhibitor protein, may play an important role in this process, as this protein can negatively regulate NF-κB during oxidative stress (13). Additionally, it is important to understand if other *Leishmania* species induce the IERSR branches, and the role, if any, in pathogenesis. Experiments carried out by our group observed an induction of the XBP-1 spliced form in clinical samples from patients infected with *Leishmania braziliensis*, another *Leishmania* species widely found in Brazil and the main causative agent of cutaneous leishmaniasis. These data indicate that other *Leishmania* species can activate this pathway, and that IERSR may play a role in *Leishmania*-associated pathogenesis.

## The Induction of ER Stress: The Role of TLRs in XBP-1 Activation in *Leishmania* Infection

The mechanism through which *L. amazonensis* induces ER stress is not understood. *Leishmania* parasitophorous vacuoles interact continuously with the ER compartment and may recruit components that are important for parasite intracellular survival (14). The inhibition of such membrane compartment fusion with the parasitophorous vacuole results in the reduction of infection (15). It is conceivable that such compartment fusions may favor the activation of IERSR branches in infection.

The contribution of TLR receptors in IERSR remains to be elucidated. There is evidence to suggest that TLRs play a role for the success of *L. amazonensis* infection that is linked with IERSR activation. For instance, when TLR2 KO macrophages were treated with the ER stress inductor thapsigargin, there was a reduction of the *L. amazonensis* proliferation compared to wild-type cells (10). Additional results obtained by our group showed that TLR2 was partially required for XBP-1 activation (splicing) due to *L. amazonensis* infection. However, the mechanism by which *L. amazonensis* induces XBP-1 activation and ER stress remains unclear.

## The PERK/ATF4 Branch of IERSR and *Leishmania* Infection: Is a Functional Role?

The PERK/ATF4 branch of IERSR plays an important role in certain cellular processes that are also exploited to establish *Leishmania* infection. For instance, *L. amazonensis* induces the PI3K/AKT signaling pathway (16), and it has been reported that the PERK-eIF2α pathway and PI3K signaling increases ATF4 expression, nuclear localization, and transcriptional activity (17–19). Additionally, PERK can directly regulate the activation of the nuclear factor (erythroid-derived 2)-like 2 (NRF2), an important antioxidant transcription factor that regulates the expression of a number of antioxidative response genes (20). Additionally, ATF4 has an important role in the autophagy. PERK/eIF2α/ATF4 signaling can induce upregulation of cytoprotective autophagy genes, such as ATG5 and ATG7, which promote cellular survival (21). In addition, ATF4 controls the microtubule associated protein 1A/1B-light chain 3 (LC3) expression. LC3B is important to generate the autophagosome formation, a hallmark of the autophagic process (22, 23). In 2012, Cyrino et al. showed that *Leishmania* parasites induce LC3B conversion and suggested that autophagy favors *L. amazonensis* infection (24). ATF4 is upregulated by HIV-1 infection and enhances HIV replication, likely due to synergistic interactions with the HIV Tat protein. Importantly, the expression of ATF4 induces HIV reactivation in chronically infected cell lines (7). Recently, our group showed that the Tat viral protein also increases *L. amazonensis* infection, in a PKR-dependent manner (25). *L. amazonensis* is able to induce PKR, a pathway activated in viral infections (26). *L. amazonensis* can also modulate IFN-1 expression in a TLR2/PKR-dependent fashion to promote the infection by the parasite, another pathway that is shared in viral infections (27). Taken together, due to classical function of IERSR in viral infections, it is relevant to test the role of PERK/ATF4 in viral co-infection and *Leishmania*.

## Conclusion Remarks

**Figure 1:**
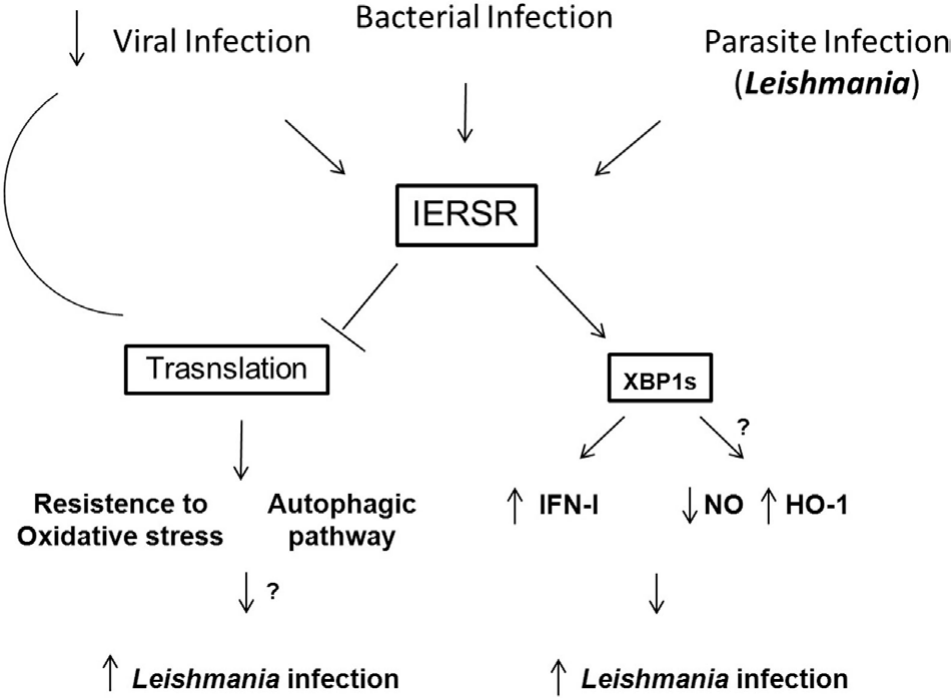
**Schematic model of the IERSR activation during *Leishmania* infection**. Viral, bacterial, and parasite infections can activate the IERSR. Viral infections can induce the activation of IERSR and lead to the inhibition of the translational process that suppress the viral replication. In *Leishmania* infection, the activation of IERSR can induce the IFN-1 production, which favors the intracellular parasite growth. Additionally, XBP1s activation is important to promote the induction of HO-1 expression, promoting the parasite infection. On the other hand, *Leishmania* infection can be favored by the control of the antioxidative response and induction of autophagic process. Both processes can be induced by the activation of IERSR, and we may predict that *Leishmania* can trigger these pathways.

It is well known that the IERSR can modulate viral and bacterial infection, promoting the induction of cytokines, including IFN-1, which can be determinant to the outcome of several infections. Recent work suggests that the IERSR is required for the development of intracellular parasites. For instance, the activation of XBP-1 in hepatocytes infected by *P. berghei* favor the infection by the parasite through the modulation of lipid synthesis. Corroborating this notion, it has been demonstrated that *L. amazonensis* activates XBP-1 leading to IFN-1 expression and the expression of antioxidative responsive genes, such as HO-1. Unveiling the mechanisms by which IERSR promote intracellular parasitic infection requires further investigation. These investigations would include determining the role of XBP-1 in resistance to oxidative stress due to *Leishmania* infection and examining other components of the ER stress signaling pathway, such as ATF6, in the context of parasitic infection. We can predict that the investigation of IERSR in intracellular parasitic infections may reveal novel drug targets. Figure 1 shows the a schematic model of IERSR activation in *Leishmania* infection.

## Author Contributions

UL – project supervisor and wrote the paper, KD-T – performed experiments and wrote the paper, RP – revised the paper and discussed the results, JS – provided samples and supervised experiments, NF – revised the paper and contributed with the discussion, BA – supervised experiments, revised the paper, and discussed results.

## Conflict of Interest Statement

The authors declare that the research was conducted in the absence of any commercial or financial relationships that could be construed as a potential conflict of interest.

## References

[1] Hetz C. The unfolded protein response: controlling cell fate decisions under ER stress and beyond. Nat Rev Mol Cell Biol (2012) 3(2):89–102. 10.1038/nrm3270 22251901

[2] Martinon F Glimcher LH. Regulation of innate immunity by signaling pathways emerging from the endoplasmic reticulum. Curr Opin Immunol (2011) 23(1):35–40. 10.1016/j.coi.2010.10.016 21094031 PMC3042531

[3] Martinon F Chen X Lee AH Glimcher LH. TLR activation of the transcription factor XBP1 regulates innate immune responses in macrophages. Nat Immunol (2010) 11(5):411–8. 10.1038/ni.1857 20351694 PMC3113706

[4] Zhang C Bai N Chang A Zhang Z Yin J Shen W ATF4 is directly recruited by TLR4 signaling and positively regulates TLR4-trigged cytokine production in human monocytes. Cell Mol Immunol (2013) 10(1):84–94. 10.1038/cmi.2012.57 23241898 PMC4003182

[5] El-Hage N Luo G. Replication of hepatitis C virus RNA occurs in a membrane-bound replication complex containing nonstructural viral proteins and RNA. J Gen Virol (2003) 84:2761–9. 10.1099/vir.0.19305-0 13679611

[6] Lloyd RE. Translational control by viral proteinases. Virus Res (2006) 119:76–88. 10.1016/j.virusres.2005.10.016 16303201 PMC7173276

[7] Caselli E Benedetti S Gentili V Grigolato J Di Luca D. Short communication: activating transcription factor 4 (ATF4) promotes HIV type 1 activation. AIDS Res Hum Retroviruses (2012) 28(8):907–12. 10.1089/AID.2011.0252 22050711

[8] Inácio P Zuzarte-Luís V Ruivo MT Falkard B Nagaraj N Rooijers K Parasite-induced ER stress response in hepatocytes facilitates Plasmodium liver stage infection. EMBO Rep (2015) 16(8):955–64. 10.15252/embr.201439979 26113366 PMC4552488

[9] Itoe MA Sampaio JL Cabal GG Real E Zuzarte-Luis V March S Host cell phosphatidylcholine is a key mediator of malaria parasite survival during liver stage infection. Cell Host Microbe (2014) 16(6):778–86. 10.1016/j.chom.2014.11.006 25498345 PMC4271766

[10] Dias-Teixeira KL Calegari-Silva TC Dos Santos GR Vitorino Dos Santos J Lima C Medina JM The integrated endoplasmic reticulum stress response in *Leishmania amazonensis* macrophage infection: the role of X-box binding protein 1 transcription factor. FASEB J (2016) 30(4):1557–65. 10.1096/fj.15-281550 26678450 PMC7163978

[11] Calegari-Silva TC Pereira RM De-Melo LD Saraiva EM Soares DC Bellio M NF-kappaB-mediated repression of iNOS expression in *Leishmania amazonensis* macrophage infection. Immunol Lett (2009) 127(1):19–26. 10.1016/j.imlet.2009.08.009 19712696

[12] Kitamura M Hiramatsu N. The oxidative stress: endoplasmic reticulum stress axis in cadmium toxicity. Biometals (2010) 23(5):941–50. 10.1007/s10534-010-9296-2 20130962

[13] Nakajima S Kitamura M Bidirectional regulation of NF-κB by reactive oxygen species: a role of unfolded protein response. Free Radic Biol Med (2013) 65:162–74. 10.1016/j.freeradbiomed.2013.06.020 23792277

[14] Ndjamen B Kang BH Hatsuzawa K Kima PE. *Leishmania* parasitophorous vacuoles interact continuously with the host cell’s endoplasmic reticulum; parasitophorous vacuoles are hybrid compartments. Cell Microbiol (2010) 12(10):1480–94. 10.1111/j.1462-5822.2010.01483.x 20497181 PMC2974788

[15] Canton J Ndjamen B Hatsuzawa K Kima PE. Disruption of the fusion of *Leishmania* parasitophorous vacuoles with ER vesicles results in the control of the infection. Cell Microbiol (2012) 14(6):937–48. 10.1111/j.1462-5822.2012.01767.x 22309219

[16] Calegari-Silva TC Vivarini ÁC Miqueline M Dos Santos GR Teixeira KL Saliba AM The human parasite *Leishmania amazonensis* downregulates iNOS expression via NF-κB p50/p50 homodimer: role of the PI3K/Akt pathway. Open Biol (2015) 5(9):150118. 10.1098/rsob.150118 26400473 PMC4593669

[17] Cao H Yu S Yao Z Galson DL Jiang Y Zhang X Activating transcription factor 4 regulates osteoclast differentiation in mice. J Clin Invest (2010) 120:2755–66. 10.1172/JCI42106 20628199 PMC2912190

[18] Inageda K. Insulin modulates induction of glucose-regulated protein 78 during endoplasmic reticulum stress via augmentation of ATF4 expression in human neuroblastoma cells. FEBS Lett (2010) 584:3649–54. 10.1016/j.febslet.2010.07.040 20667453

[19] Lian N Lin T Liu W Wang W Li L Sun S Transforming growth factor β suppresses osteoblast differentiation via the vimentin activating transcription factor 4 (ATF4) axis. J Biol Chem (2012) 287:35975–84. 10.1074/jbc.M112.372458 22952236 PMC3476265

[20] Dey S Sayers CM Verginadis II Lehman SL Cheng Y Cerniglia GJ ATF4-dependent induction of hemeoxygenase 1 prevents anoikis and promotes metastasis. J Clin Invest (2015) 125(7):2592–608. 10.1172/JCI78031 26011642 PMC4563676

[21] Luo J-Q Chen D-W Yu B. Upregulation of amino acid transporter expression induced by l-leucine availability in L6 myotubes is associated with ATF4 signaling through mTORC1-dependent mechanism. Nutrition (2013) 29:284–90. 10.1016/j.nut.2012.05.008 22985970

[22] Kabeya Y Mizushima N Ueno T Yamamoto A Kirisako T Noda T LC3, a mammalian homologue of yeast Apg8p, is localized in autophagosome membranes after processing. EMBO J (2000) 19(21):5720–8. 10.1093/emboj/19.21.5720 11060023 PMC305793

[23] Baehrecke EH. Autophagy: dual roles in life and death? Nat Rev Mol Cell Biol (2005) 6(6):505–10. 10.1038/nrm1666 15928714

[24] Cyrino LT Araújo AP Joazeiro PP Vicente CP Giorgio S. In vivo and in vitro *Leishmania amazonensis* infection induces autophagy in macrophages. Tissue Cell (2012) 44(6):401–8. 10.1016/j.tice.2012.08.003 22939777

[25] Vivarini Áde C Pereira Rde M Barreto-de-Souza V Temerozo JR Soares DC Saraiva EM HIV-1 Tat protein enhances the intracellular growth of *Leishmania amazonensis* via the ds-RNA induced protein PKR. Sci Rep (2015) 5:16777. 10.1038/srep16777 26608746 PMC4660360

[26] Pereira RM Teixeira KL Barreto-de-Souza V Calegari-Silva TC De-Melo LD Soares DC Novel role for the double-stranded RNA-activated protein kinase PKR: modulation of macrophage infection by the protozoan parasite *Leishmania*. FASEB J (2010) 24(2):617–26. 10.1096/fj.09-140053 19812373

[27] Vivarini Ade C Pereira RM Teixeira KL Calegari-Silva TC Bellio M Laurenti MD Human cutaneous leishmaniasis: interferon-dependent expression of double-stranded RNA – dependent protein kinase (PKR) via TLR2. FASEB J (2011) 25(12):4162–73. 10.1096/fj.11-185165 21846836

